# Use of the Normalized Difference Road Landside Index (NDRLI)-based method for the quick delineation of road-induced landslides

**DOI:** 10.1038/s41598-018-36202-9

**Published:** 2018-12-13

**Authors:** Yinjun Zhao, Yuying Huang, Hanhu Liu, Yongping Wei, Qing Lin, Yuan Lu

**Affiliations:** 1 Key Laboratory of Environment Change and Resources Use in Beibu Gulf, Ministry of Education, Guangxi Teachers Education University, 175 Mingxiu East St., Nanning, 530001 China; 2 School of Geography and Planning, Guangxi Teachers Education University, 175 Mingxiu East St., Nanning, 530001 China; 3 College of Earth Sciences, Chengdu University of Technology, Chengdu, 610059 China

## Abstract

Recognition and classification of road-related landslides are a critical requirement in pre- and post-disaster hazard analysis. They are primarily done through field mapping or manual image interpretation from commercial satellites images. This paper developed a Normalized Difference Road Landside Index (NDRLI)-based method to delineate road-induced landslides and enhance their presence in remotely sensed digital imagery based on free Landsat Operational Land Imager (OLI) sources. The NDRLI-based method includes NDRLI, Shape Index of Spectral Curve (SISC), and other optimizing steps such as deleting shadow and slope <20° area to recognise landslides. The test results show that the NDRLI-based method is effective in extracting road-induced landslide information, although the Kappa coefficient should be further improved.

## Introduction

**Figure 1:**
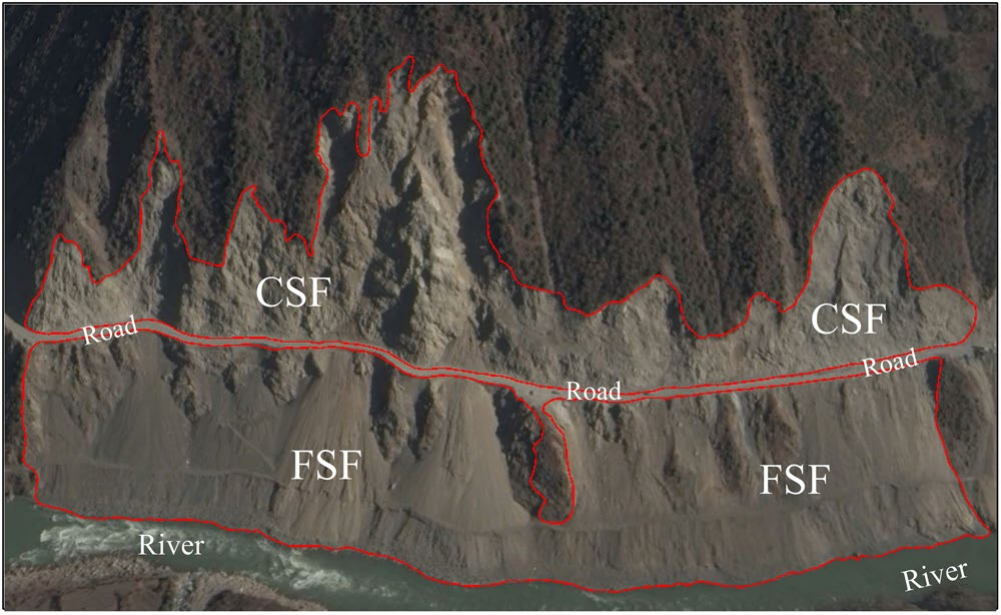
Typical road-induced landslides: fillslope failure (FSF) and cutslope failure (CSF) are their main interpretation keys of remote sensing. Both main scarps of FSF and CSF are relatively obvious border and brighter than surrounding colour. From image texture, we can see the traces and stacks caused by movement of the displaced material and surface erosion. The vegetation coverage is relative low. The CSF with excavation signs always locate above and adjacent roads with steep slope, and the FSF always locate under and adjacent roads with relative big slope. The image was provided by DigitalGlobe and obtained from Google Earth 7.1.

Roads constitute an archetypical example of the tension between human development and environment degradation, especially in mountainous areas, as they are necessary for the former and can lead directly to the latter^1^. Landslides are one of the world’s worst natural disasters and lead to loss of life and property, blocking of roads and rivers, disruption of communication and triggering of floods^2^. Based on major disasters between 1980 and 2002, 640 persons in Colombia in 1987, 472 persons in Nepal in 2002 and 400 persons in India in 1995 were killed by slides, including avalanches and landslides, while Nepal suffers 116.25 average deaths per landslide and 71.41 in China^3^. Many landslides are derived from or related to road networks^4^. In particular, mountain roads are the most prodigious source of landslide sediments of all widespread land uses^5^. During road construction, roads are embedded into or through steep hillsides by blasting and excavating, which can create large areas of instability by the use of cut- and fill- construction. Cutting into hillsides and then removal of the toe of slopes or filling slopes to widen and reinforce roads both effectively reduce the slope cohesion and strength, and contribute to slope failures (Fig. 1). On the down slope side of built roads, stack areas of discarded material from road construction are common. Moreover, road construction interrupts surface drainage, ditches and culverts, and alters subsurface water movement, changes the distribution of mass and increase erosion because of road-related deforestation and construction activities^2,5^. All of the above factors could facilitate landslides during and after road construction. For example, the volume of slide material in the western Cascade Range, Oregon, removed from road right-of-way has been 65470 m^3^/km^2^, which is 30 times the rate of slide activity in undisturbed forested areas^6^. Unprecedented rates of landslides and surface erosion were noted after the construction of Weixi-Shangri road (23.5 km) in Yunan province, China. These rates averaged up to 9600 t ha^−1^ ^5^.

However, along with social and economic development, people in mountainous areas who are always poor have a strong desire to be wealthy. Constructing transportation networks is usually the first and key step to support tourism, trade, agricultural development and local travel. Thus, considerable scientific literature has been published regarding the development of a landslide inventory, including susceptibility^7–10^ and hazard zoning for land-use planning, avoiding landslide-prone areas, engineering design^11^ and developing efficient ways to reduce future damage, but only a few studies have directly focused on road-induced landslides^4,12–15^.

Based on the development of computer and satellite technologies, Remote Sensing (RS) techniques have been widely used in landslide studies and are well suited to acquire and analyse spatial data related to landslides^16^. Satellite imagery offers an economical and fast method to monitor and map landslides over large and inaccessible areas. The last few decades have witnessed the increasing use of RS techniques, such as interpretation of aerial photography, stereoscopic image analysis, interferometry studies, and Light Detection and Ranging (LiDAR) for identifying, detecting, monitoring, cataloguing, assessing risk, and mapping^17–22^, but few studies have reported the automated methods for extracting road-related landslide inventories^16,23^. Borghuis^24^ showed how unsupervised classification could detect 63% of all landslides mapped manually. The automated and semi-automated methods can improve working efficiency compared to time-consuming visual interpretations, which are fraught with the subjectivity of the visual interpreters, and lower workload^19,20^.

Therefore, in view of huge successes using Normalized Difference Vegetation Index (NDVI) and free Landsat Operational Land Imager (OLI) sources, our study aimed at designing a new remote sensing index, Normalized Difference Road Landside Index (NDRLI), in conjunction with object-oriented classification methods using Landsat OLI satellite images and digital elevation model (DEM) derived slopes to automatically classify road-induced landslide (i.e., cutslope and fillslope failures resulting from cut-construction and fill-construction, respectively) locations and area. The NDRLI-based method was tested in Yunnan province, southwest China, the most landslide-prone area in the country, which has experienced or is currently experiencing extensive roads construction. This method is a new solution for mapping landslides for road management and other relevant applications.

## Results

**Figure 2:**
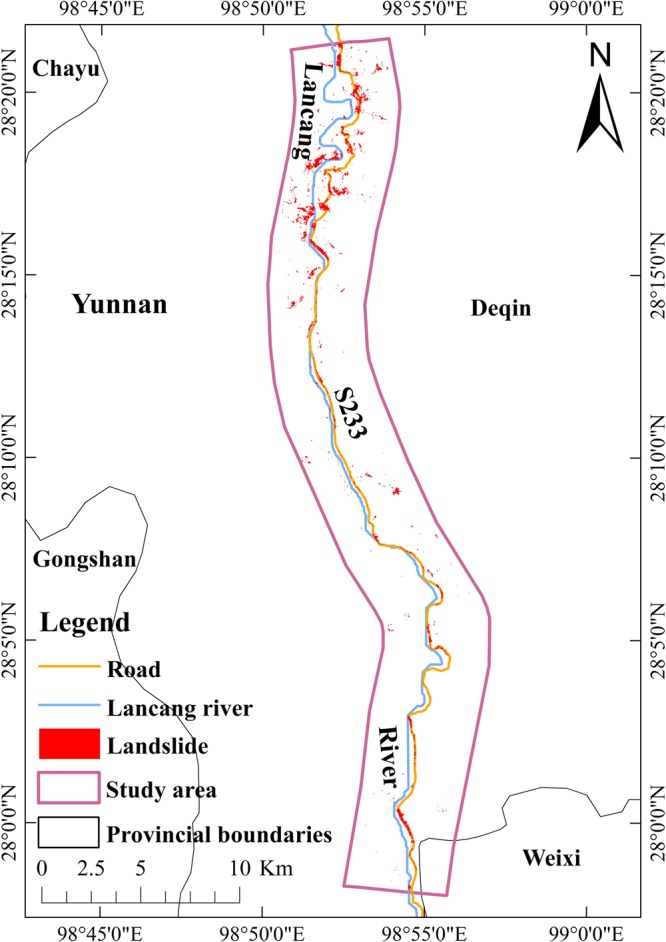
The distribution of road-induced landslides from Normalized Difference Road Landside Index (NDRLI)-based method based on Landsat 8 OLI data^26^. The map was generated using ArcGIS10.1 (http://www.esrichina.com.cn/softwareproduct/ArcGIS/).

According to NDRLI-based, road-induced landslide classification method, described in Materials and Proposed NDRLI-based method section, we calculated the NDRLI to classify potential road-induced landslides and the Shape Index of Spectral Curve (SISC) to reduce bare soil and farmland noise. Then, we removed shadow areas using object-oriented classification methodology and further reduced farmland by using the angle of slope rule. The entire process was performed using ENVI, ArcGIS and Google Earth. The final classification of road-induced landslides was shown in Fig. 2. The total area of road-induced landslides in study area is 4.38 km^2^ and accounts for 4.47% of the total study area.

The accuracy of classification is the primary issue for the application of NDRLI-based methods in many fields, including road planning and risk assessment. Therefore, it is necessary to evaluate the performance of NDRLI-based methods. We sampled some validated areas from Google Earth as real surface regions of interest to calculate a confusion matrix. Precision evaluation was performed for the classified images by using the confusion matrix in image processing software. The overall accuracy is 98.49%, and the Kappa coefficient is 0.51, which belongs to moderate level. By comparison, error classification increases with the distance to roads. Previous research has shown that the distance from roads increases the landslides constituting declines and landslides usually occurred at the distance range of 0~50 m^25^. In addition, Fig. 2 also clearly shows that most landslides are near the S233 road and the accuracy will decrease with the increase of distances to roads. The Kappa coefficient increased to 0.74 when we reduced our study area to a 100 m buffer area along the road.

## Conclusion and Discussion

The NDRLI-based method is new method that has been developed primarily to extract road-induced landslides and to enhance their presence in remotely sensed, digital imagery, while simultaneously removing bare soil, farmland, water and vegetation features in cooperation with other information. This new method can quickly and efficiently discriminate road-induced landslides from background. Noise from vegetation, water, bare soil and farmland can be reduced and even removed. This method is automatic and would be very useful for large regions, especially low accessibility mountainous areas. In a non-road situation, the landslides caused by human engineering activity may be extracted by the NDRLI-based method because of their similarity with road-induced landslides, but the accuracy is probably lower due to the increase of misclassification with the distance to roads.

**Figure 3:**
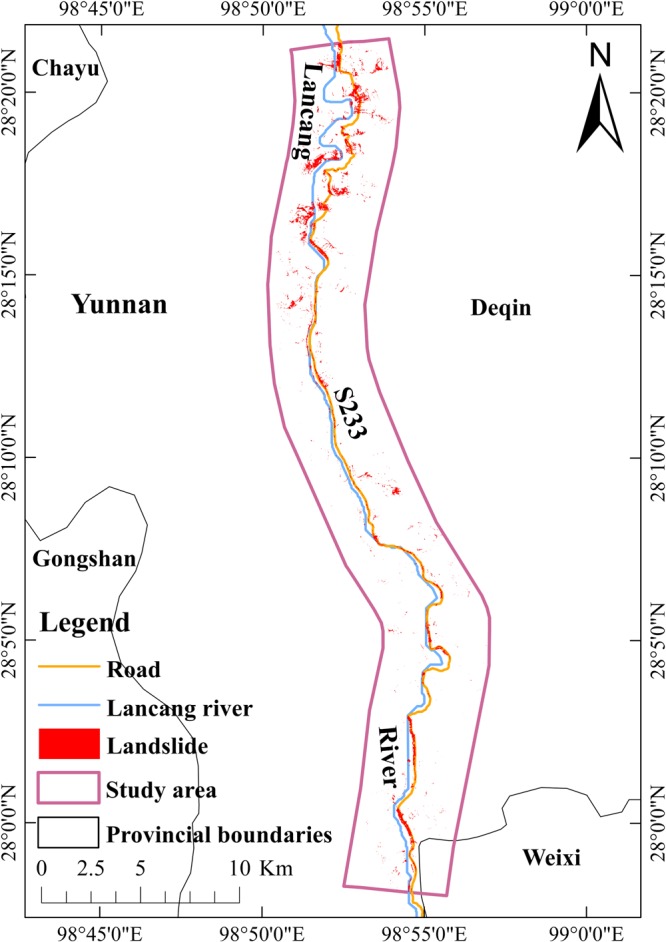
The distribution of road-induced landslides from NDRLI-based method based on Sentinel-2 data.

Sentinel-2 sensor is very similar to Landsat-8 at least in the bands 1–7 used in this study, and has the advantage in spatial resolution over Landsat 8. We firstly assumed that Sentinel-2 images will perform well in the NDRLI-based method, and then the adaptation was done step by step. Sentinel-2A data of the study area (20 m and 10 m spatial resolution) from European Space Agency (https://scihub.copernicus.eu/) were downloaded and pre-processed. The NDRLI was made the corresponding changes (Equation 1) according to the band differences. The SISC used 1.15 as the threshold value obtained through the trial-and-error attempts described in Optimizing results section. After that, the road-induced landslides were extracted from Sentinel-2A. Moreover, we build a 7.5 m buffer according to actual road width along a digitized centreline of provincial road S233 in Google Earth to delete the road noises due to the coarse resolution of Shuttle Radar Topography Mission (SRTM) DEMs. Finally, the area of road-induced landslides was identified to be 5.52 km^2^ which accounts for 5.63% of the total study area (Fig. 3). The overall accuracy is 94.63%, and the Kappa coefficient is up to 0.81. Compared to Landsat results, the landslide areas and Kappa coefficient have increased by 1.14 km^2^ and 0.07, respectively, mainly due to the increase of spatial resolution of Sentinel-2A. The overlap area of these two results accounts for 70.3% of total landslides area derived from Landsat-8, while the unoverlap area mostly comes from small landslides derived from Sentinel-2.

1 $${\rm{NDRLI}}=\frac{SWIR-BLUE}{SWIR+BLUE}$$

There are still several aspects for further study. First, terrain shadow is an important impact factor on classification accuracy. Extracting landslides from shadow OLI images is very difficult, so our method didn’t consider this situation. Second, misclassification of bare soil and farmland still exists. If the threshold value of NDRLI is appropriate, the noise could be removed. These thresholds should be tested to find suitable values for different areas. Third, each pixel of Landsat 8 OLI image is up to 900 m^2^, so the edges of landslide pixel may have other features. Mixed pixels will reduce classification accuracy. Fourth, we used Google Earth images as the ground truth map of the landslides without field investigation^21^ or using LiDAR^22^, which might be slightly unreliable.

Although Kappa coefficient is not high, we still believe that this study will be meaningful because of the mixed characteristics of landslides. In the future, shadow effect can be overcome by collaboration with shadow enhancement and detection, and high resolution images. Using other features (e.g., texture) are directions to try. This method could provide insights for further studies.

## Materials and Proposed Ndrli-Based Method

### Study area and data

**Figure 4:**
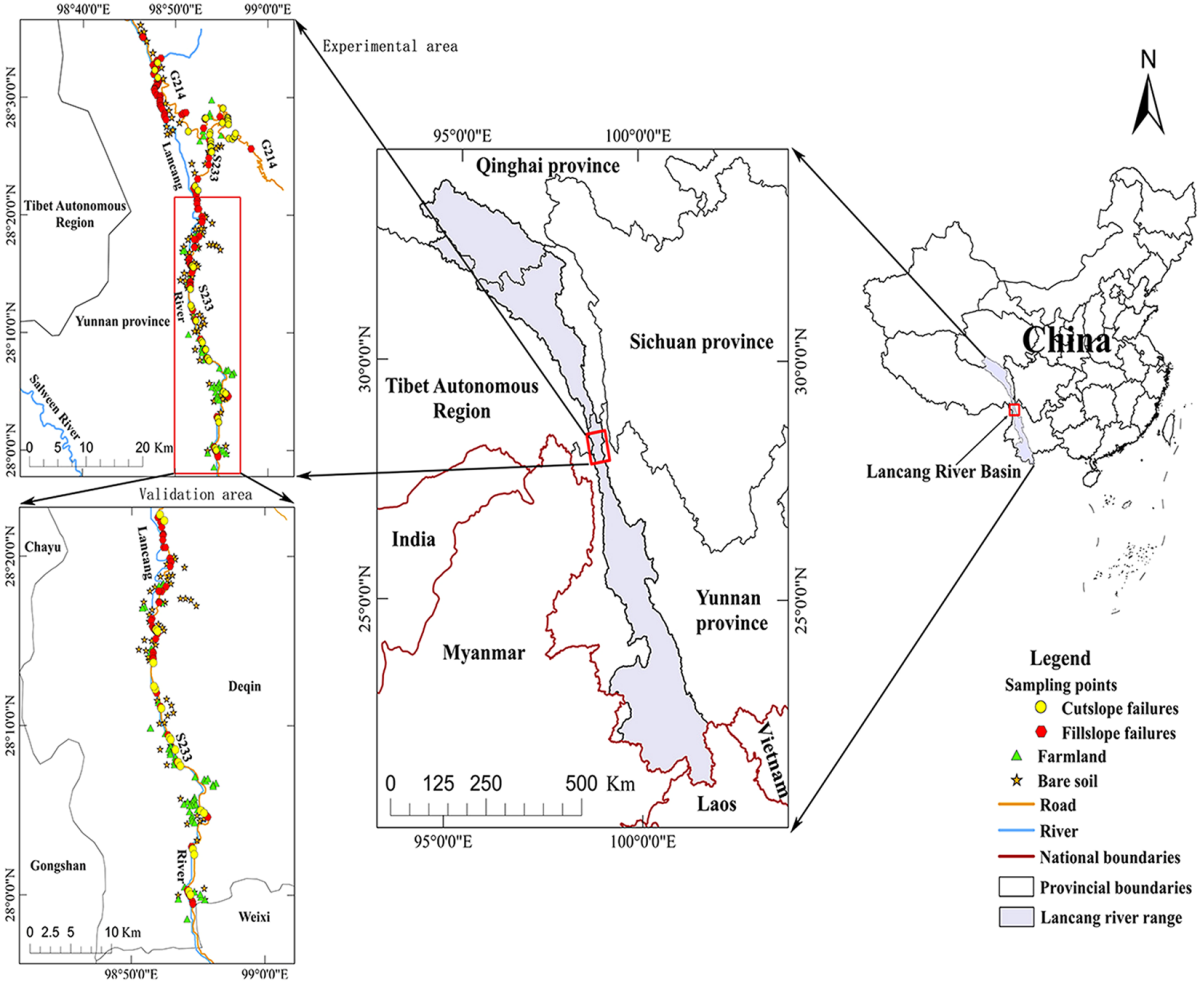
Locations and basic information of the study area, generated by ArcGIS10.1 (http://www.esrichina.com.cn/softwareproduct/ArcGIS/). The length of the S233 in the validation area is 49 km.

The Lancang River runs through the Hengduan Mountains with its complex landforms. This area is known as a remote mountainous region and is the most landslide-prone area in the country, especially in the midstream region. Approximately 1184 km of roads, including highways, national roads and provincial roads, were built in the midstream region with its steep mountains and deep valleys. The road density is extremely high. Thus, we selected a section of S233 (a provincial road) and G214 (a national road) along the middle of the Lancang River as targets to build a 2 km wide road buffer to establish our study areas (Fig. 4).

Landsat 8 OLI images (24-12-2014, Path 132/ Row 041) were downloaded from the United States Geological Survey (http://glovis.usgs.gov/)^26^. SRTM DEMs with 30 m spatial resolutions were generated by National Aeronautics and Space Administration and provided by the Data Center for Resources and Environmental Sciences, Chinese Academy of Sciences (RESDC) (http://www.resdc.cn). Google Earth was chosen as the major data source to extract and validate road-related landslide as it allows users to obtain free high-resolution satellite images from around the world and to measure the length and height of target objects. It becomes a very popular and reliable data source or an additional option in many studies^27–29^. For example, Goudie^27^ used Google Earth to quantify pan and creek characteristics of salt marshes on Google Earth imagery at 100 × 100 m scale.

### Technical framework

**Figure 5:**
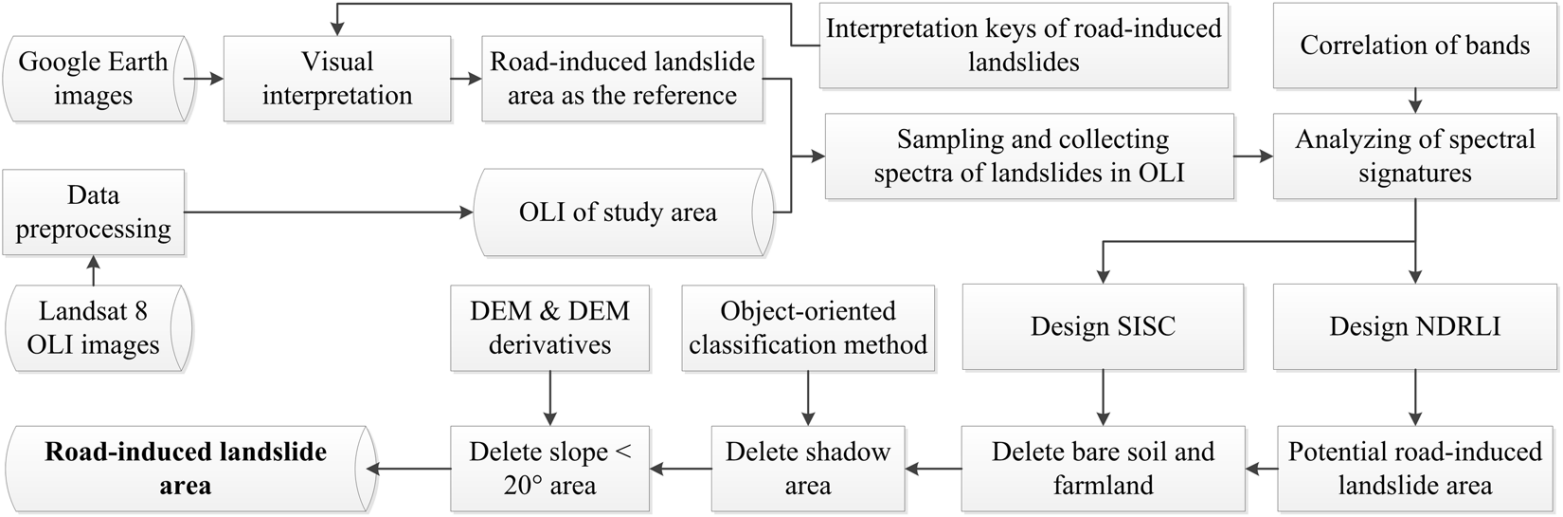
Workflow for NDRLI-based road-induced landslides classification.

NDRLI-based methodology includes three major stages: sampling road-induced landslides; designing RS indexes; and optimizing results to eliminate other mixed types as shown in Fig. 5. In the first stage, the locations and areas of cutslope and fillslope failures along roads were depicted and randomly sampled using Google Earth by visual interpretation according to interpretation keys (Fig. 1). To reduce the impact of moisture on landslide interpretation results, images during drought periods are good options because the landslide surfaces were often mixed soil, rock, and detrital grain. Landsat OLI satellite images were pre-processed, including radiometric calibration, atmospheric correction and image cutting. For the second stage, we identified the location of known, road-induced landslides and then sampled spectral signatures at the same locations in OLI images. Plotting spectrum curves and analyzing the correlation of 7 bands of OLI images provided some different spectral laws between landslides and other surface features that helped to develop a new RS index method (NDRLI). In the final stage, other mixed surface features like bare land from potential road-induced landslide region were eliminated to optimize final landslide area.

### Designed NDRLI

**Figure 6:**
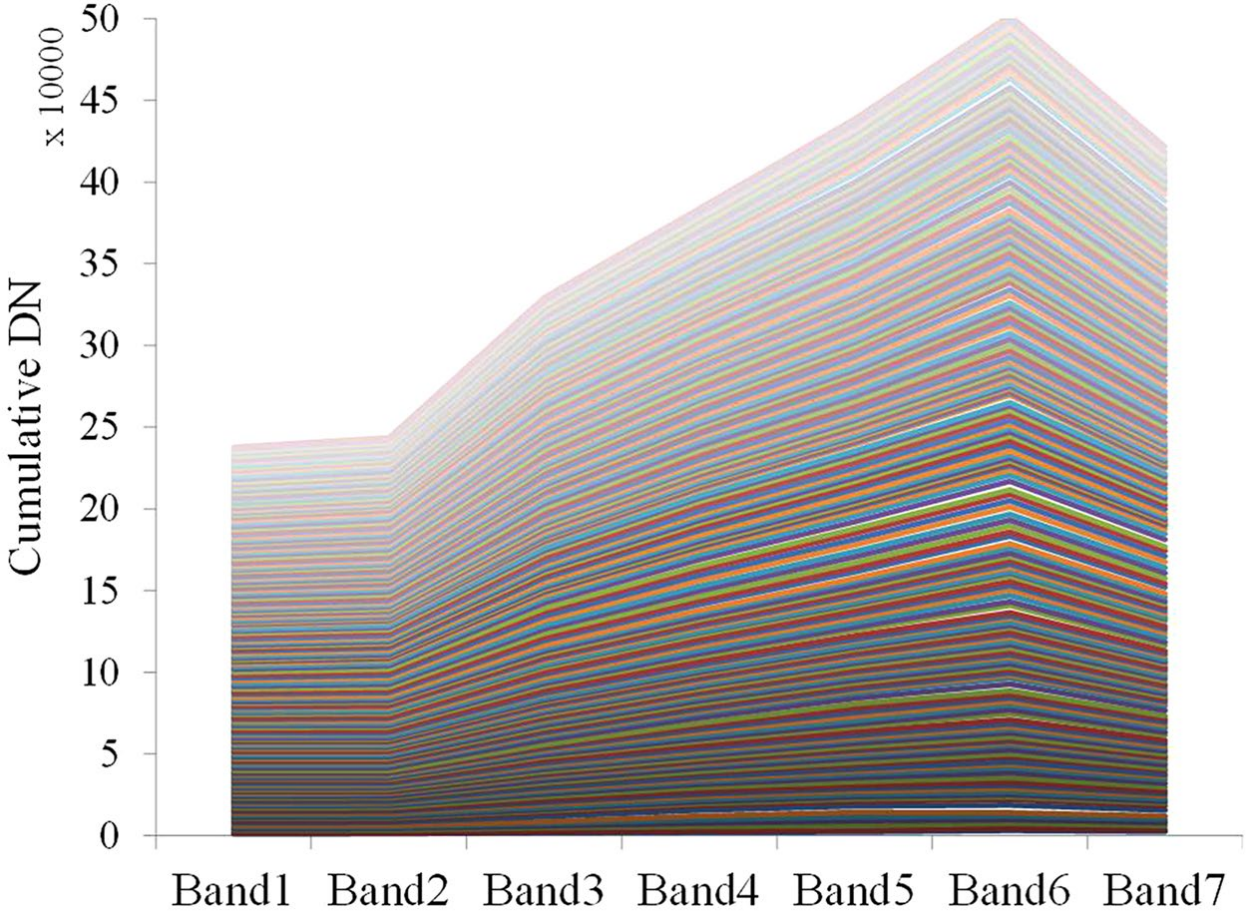
Cumulative spectrum curves of road-induced landslides (n = 194).

According to Fig. 5, 194 samples, including 155 fillslope and 39 cutslope failures, were depicted along S233 and G214 road in Google Earth. Spectrum curves of these points were then plotted in Fig. 6. Spectrum curves are mainly determined by material composition, such as soil, sand and rock, and are affected by moisture.

Figure 6 clearly shows that reflectance of major road-induced landslides reaches its maximum level at band 6 and its minimum level at band 2. Ground objects with high temperatures typically have a high shortwave infrared (SWIR) reflectance. Thus, landslides primarily covered by bare soil, gravel and detrital grain have a higher reflectance at SWIR than other objects at ambient temperatures (e.g., vegetation, water, soil) because of the landslide surface has a higher temperature. Spectrum curves of landslide peak magnitudes based on SWIR (1.6 μm) were at a minimum at the Blue band (0.45 μm). While band 6 is better in identifying bare soil and low water content areas, band 2 is better at identifying soil and vegetation.

To find further differences among the 7 bands, the principal component analysis between bands was conducted after atmospheric correction of the images. According to the covariance matrix (Tables 1 and 2), band 6 has the maximal covariance with other bands, which means that band 6 could represent more information. Table 2 shows that near-infrared reflectance (NIR) band has relatively better independence than other bands in general, and next is band 2 and band 6. The correlation coefficient between band 2 and band 6 is 0.84 that is the second smallest. Band 6 is carrier of with ample information.

**Table 1:** The covariance matrix of each band of Landsat 8 × 10^3^.

Covariance	Band 1	Band 2	Band 3	Band 4	Band 5	Band 6	Band 7
Band 1 (Coastal)	88.28						
Band 2 (Blue)	87.64	88.02					
Band 3 (Green)	115.82	117.15	163.22				
Band 4 (Red)	138.08	140.77	197.88	245.28			
Band 5 (NIR)	227.75	230.87	352.55	434.57	1051.28		
Band 6 (SWIR1)	261.01	267.28	395.52	499.86	1029.84	1142.92	
Band 7 (SWIR2)	195.94	200.48	290.08	365.87	698.80	805.75	580.92

**Table 2:** The correlation matrix of each band of Landsat 8.

Correlation	Band 1	Band 2	Band 3	Band 4	Band 5	Band 6	Band 7
Band 1 (Coastal)	1.00						
Band 2 (Blue)	0.99	1.00					
Band 3 (Green)	0.96	0.98	1.00				
Band 4 (Red)	0.94	0.96	0.99	1.00			
Band 5 (NIR)	0.75	0.76	0.85	0.86	1.00		
Band 6 (SWIR1)	0.82	0.84	0.92	0.94	0.94	1.00	
Band 7 (SWIR2)	0.87	0.89	0.94	0.97	0.89	0.99	1.00

The band-ratio method takes advantage of the differences in the reflectance of different wavelengths of light from any given surface^30^. For example, NDVI uses the condition where the features that have higher NIR and lower red light reflectance will be enhanced, while those with low red light reflectance and very low NIR reflectance will be suppressed or even eliminated.

In conclusion, the NDRLI was designed using similar principles that were learned from NDVI. The NDRLI is calculated as follows:

2 $${\rm{NDRLI}}=\frac{{\rm{SWIR}}1-{\rm{BLUE}}}{{\rm{SWIR}}1+{\rm{BLUE}}}$$

where BLUE is blue light band, and NDRLI ranges from −1 to +1. The index is designed to (1) maximize reflectance of road-induced landslides and probable bare soil by using SWIR1, (2) minimize the low reflectance of blue light by water and (3) take advantage of the high reflectance of SWIR by road-induced and bare soil features. As a result, road-induced landslides and bare soils are enhanced, while water usually has negative values and therefore is suppressed. In addition, vegetation and farmland are also more enhanced than road-induced landslides. After many trial-and-error attempts, we found that the NDRLI range of 0 to 0.5 are probably road-induced landslide areas, around 0.4~0.7 is probably farmland and bare soil, and around 0.7~1 is probably vegetation. Thus, there are overlaps in the NDRLI thresholds among road-induced landslides, farmland and bare soil. This is consistent with the mixed characteristics of road-induced landslides.

### Optimizing results

The information of delineated, road-induced landslide was often mixed with the noise from farmland, bare soil and a little built-up land. This happens because most road-induced landslides are mixtures, and the surfaces of bare soil with lower vegetation coverage rate and pre-planting farmland are mostly bare. For example, some unstable landslides are fully or partly covered with vegetation after several years of road construction. Their reflectance pattern in the blue light band and SWIR1 is similar to that of road-induced landslides, i.e., they both reflect shortwave infrared light more than they reflect blue light. As a result, the computation of the NDRLI also produces a positive value for farmland and bare soil.

**Figure 7:**
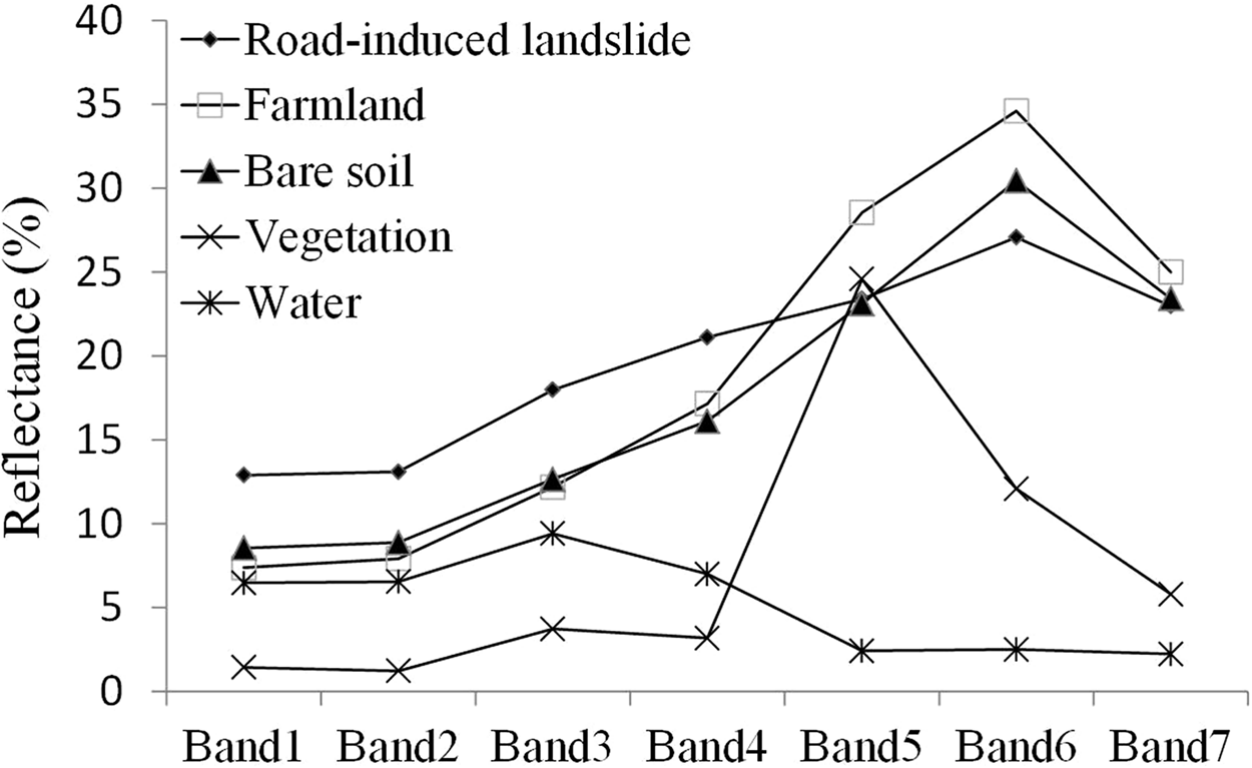
Spectral reflectance patterns of road-induced landslides (155 FSF and 39 CSF), farmland (n = 133), and bare soil (n = 99), in raw Landsat OLI satellite images^23^.

To remove the farmland and bare soil noise from NDRLI’s potential landslide area, we carefully plotted the spectral reflectance patterns of common land cover types and landslides (cutslope and fillslope failure) from the test area of this study. A detailed examination of the signatures in Fig. 7 reveals that the average reflectance derived from farmland and bare soil at bands 3 (green band), 4 (red band) and 5 (NIR band) formed concave curves, but landslides approximated convex curves. Therefore, if the mean of band 3 and band 5 is divided by band 4, farmland and bare soil should be greater than 1, but landslides, less than or equal to 1. Based on this assumption, we designed a SISC to remove noise. The SISC can be expressed as follows:

3 $${\rm{SISC}}=\frac{({\rm{GREEN}}+{\rm{NIR}})/2}{{\rm{RED}}}$$

**Figure 8:**
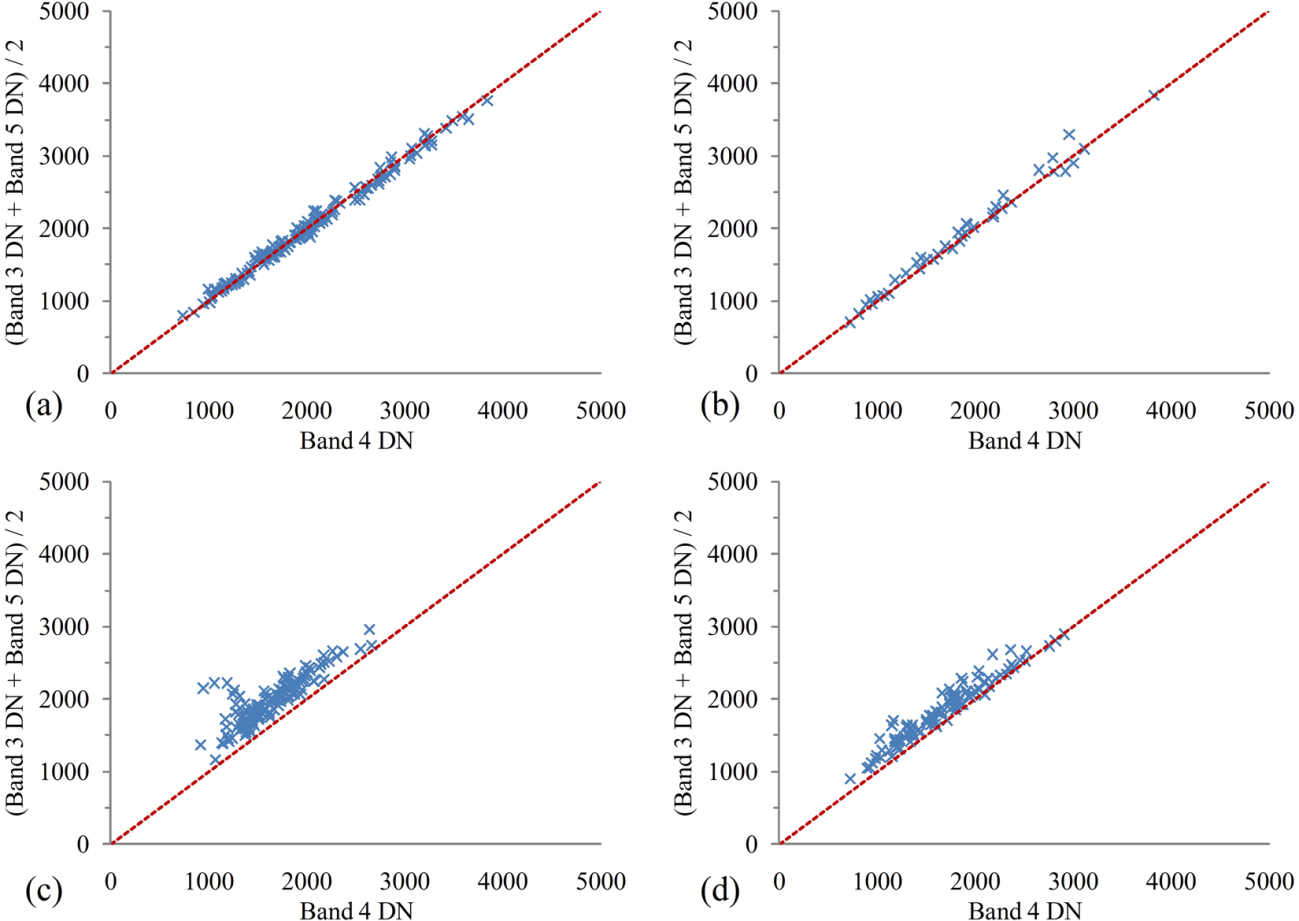
Scatter plots of the band 4 digital number (DN) with the average DN of the band 3 and band 4 (**a**) FSF (n = 155), (**b**) CSF (n = 39), (**c**) farmland (n = 133) and (**d**) bare soil (n = 99).

**Figure 9:**
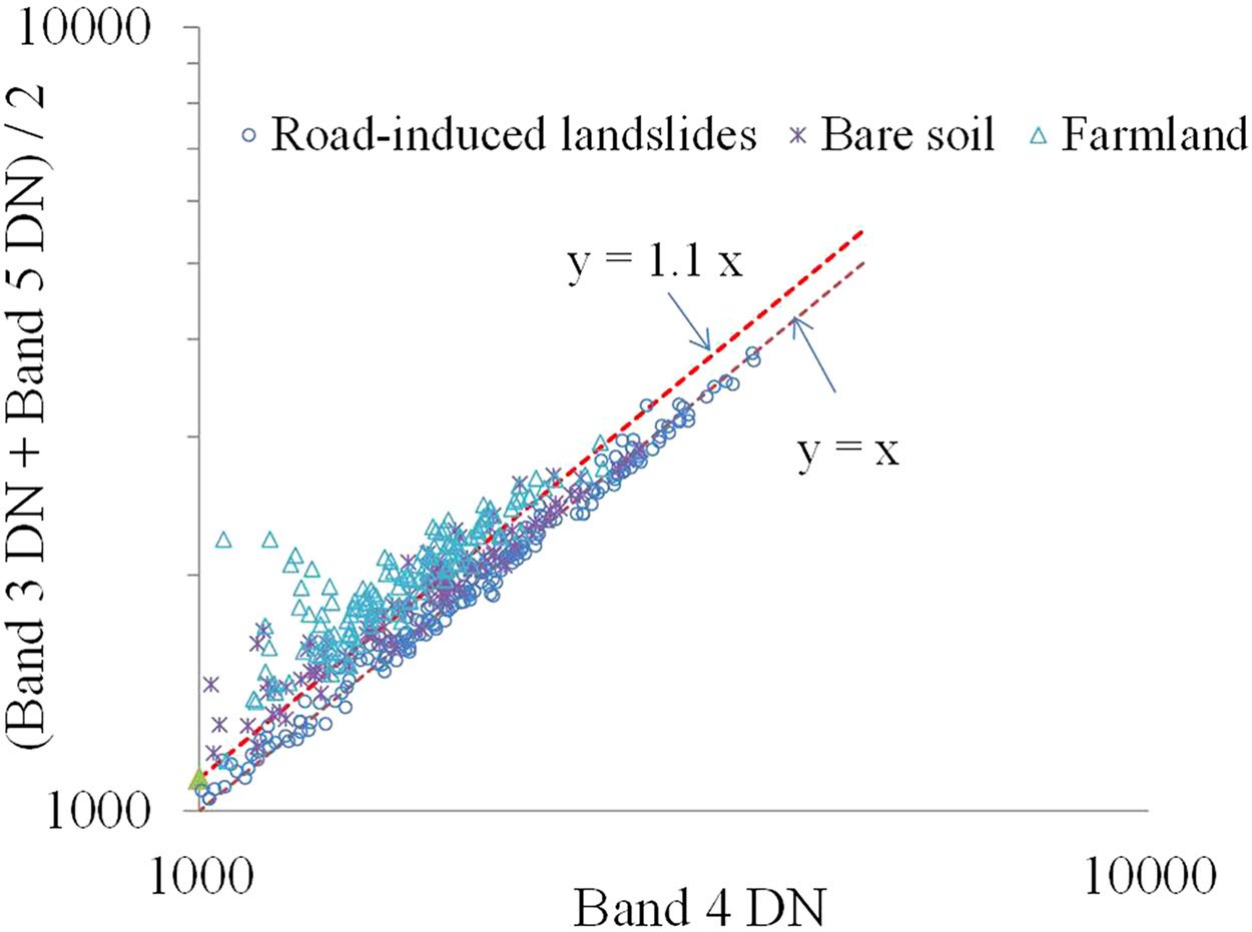
(**a**) FSF (n = 155), (**b**) CSF (n = 39), (**c**) farmland (n = 133) and (**d**) bare soil (n = 99).

To further assess the result of SISC, we plotted the scatter plots to compare the band 4 to the mean of band 3 and band 5, from the test areas of this study, as shown in Fig. 8. It is very clear that the computations of SISC for farmland and bare soil are greater than 1, while the landslide results are close to 1. In view of the fact that most road-induced landslides usually contain soil, rock, and vegetation, we have tested the threshold of SISC and, after many trial-and-error attempts (Table 3), found 1.100 to be a suitable value to remove farmland and bare soil noise, e.g., while ground objects with higher SISC values (>1.100) will be removed as noise (Fig. 9).

**Table 3:** Three test scenarios with different SISC values.

Types	Total number of samples	Scenario 1 SISC > 1.100	Scenario 2 SISC > 1.075	Scenario 3 SISC > 1.050
		Accuracy rate (%)	Accuracy rate (%)	Accuracy rate (%)
Bare soil	42	64.3	66.0	81.0
Farmland	46	89.1	89.1	89.1
FSF	84	99.0	97.6	92.9
CSF	34	95.4	82.3	58.8

Note: If SISC > 1.100, ground objects are classified into farmland or bare soil, ground objects are landslides. Same rules are for SISC > 1.075 and >1.050 scenarios.

The geometry and radiometry of satellite imagery are significantly affected by the topography of mountainous areas due to shadow effects^31^. Many classification methods fail in mountainous areas, where terrain shadow effects are wide and difficult to eliminate. The same features under terrain shadows have different spectral reflectance. Therefore, we did not consider landslides in the shadow area. We randomly selected 20 shadow and non-shadowed samples from the image after atmospheric correction for region of interest statistical analysis in ENVI 5.3. We found the smallest overlap of spectral values of each band between shadow and non-shadowed area is within the range from 0~380 in NIR band. Then spectral values from 0~380 in NIR band was used to create a classification rule for extracting shadow area with the Feature Extraction tool of ENVI 5.3. Finally, the shadow area was eliminated from the study area with an object-oriented classification method.

We also can use slope to remove farmland from potential road-induced landslides. The relationship between landslides and slope is well understood. Slope is a key factor of landslides and internal conditions that trigger landslides. Many statistics indicated that landslide-prone slope is 20°~50° ^32^. On the other hand, most farmland is located at slopes less than 15°. If farmland has a slope greater than 25°, it should be returned to forestland in China. Therefore, a slope of less than 20° was used as a threshold to further remove farmland and improve classification accuracy.

## References

[1] Bernardi De León R (2009). Road development in Podocarpus National Park: an assessment of threats and opportunities. Journal of sustainable forestry.

[2] Banerjee, P. & Ghose, M. K. Spatial analysis of environmental impacts of highway projects with special emphasis on mountainous area: an overview. *Impact Assessment & Project Appraisal* 1–15 (2016).

[3] Kahn ME (2005). The death toll from natural disasters: the role of income. geography, and institutions. Review of Economics & Statistics.

[4] Seutloali KE, Beckedahl HR (2015). A review of road-related soil erosion: an assessment of causes, evaluation techniques and available control measures. Earth Sciences Research Journal.

[5] Sidle RC, Furuichi T, Kono Y (2011). Unprecedented rates of landslide and surface erosion along a newly constructed road in Yunnan, China. Natural Hazards.

[6] Swanson F. J. Impact of clear-cutting and road construction on soil erosion by landslides in the western Cascade Range. *Oregon* (1975).

[7] Hong Y, Robert A, George H (2007). Use of satellite remote sensing data in the mapping of global landslide susceptibility. Natural Hazards.

[8] Pellicani R, Ilenia A, Giuseppe S (2017). GIS-based predictive models for regional-scale landslide susceptibility assessment and risk mapping along road corridors. Geomatics, Natural Hazards and Risk.

[9] Erener, A., Sarp, G. & Duzgun, S. H. Use of GIS and Remote Sensing for Landslide Susceptibility Mapping. In *Encyclopedia of Information Science and Technology*, Fourth Edition, 3503–3514. IGI Global (2018).

[10] Lee CF (2018). Regional landslide susceptibility assessment using multi-stage remote sensing data along the coastal range highway in northeastern Taiwan. Geomorphology.

[11] Hearn GJ, Massey CI (2009). Engineering geology in the management of roadside slope failures: Contributions to best practice from Bhutan and Ethiopia. Revista De Microbiologia.

[12] Jaafari A, Najafi A, Rezaeian J, Sattarian A, Ghajar I (2015). Planning road networks in landslide-prone areas: A case study from the northern forests of Iran. Land Use Policy.

[13] Sidle RC (2006). Erosion processes in steep terrain-truths, myths, and uncertainties related to forest management in Southeast Asia. Forest Ecol Manag.

[14] Sadek, S., Ramadan, R. & Al-Naghi, H. A GIS-based landslide hazard framework for road repair and maintenance. *Electronic Journal of Geotechnical Engineering* **10** (2007).

[15] Achour Y (2017). Landslide susceptibility mapping using analytic hierarchy process and information value methods along a highway road section in Constantine. Algeria. Arabian Journal of Geosciences.

[16] Aksoy B, Ercanoglu M (2012). Landslide identification and classification by object-based image analysis and fuzzy logic: An example from the Azdavay region (Kastamonu, Turkey). Computers & Geosciences.

[17] Tralli DM, Blom RG, Zlotnicki V, Donnellan A, Evans DL (2005). Satellite remote sensing of earthquake, volcano, flood, landslide and coastal inundation hazards. Isprs Journal of Photogrammetry & Remote Sensing.

[18] Pradhan, B. Laser Scanning Applications in Landslide Assessment. *Springer International Publishing* (2017).

[19] Hölbling D (2012). A semi-automated object-based approach for landslide detection validated by persistent scatterer interferometry measures and landslide inventories. Remote Sensing.

[20] Hölbling D, Friedl B, Eisank C (2015). An object-based approach for semi-automated landslide change detection and attribution of changes to landslide classes in northern Taiwan. Earth Science Informatics.

[21] Torkashvand AM, Irani A, Sorur J (2014). The preparation of landslide map by landslide numerical risk factor (LNRF) model and geographic information system (GIS). The Egyptian Journal of Remote Sensing and Space Science.

[22] Wiedenmann J, Rohn J, Moser M (2017). Using LIDAR and ground truth for landslide recognition and characterization of geotechnical and morphological parameters in sedimentary rocks, a case study in Northern Bavaria (Germany). Journal of Mountain Science.

[23] Price, S. R., Price, S. R., Price, C. D. & Blount, C. B. Pre-screener for automatic detection of road damage in SAR imagery via advanced image processing techniques. In *Pattern Recognition and Tracking XXIX*, 1064913. International Society for Optics and Photonics (2018).

[24] Borghuis AM, Chang K, Lee HY (2007). Comparison between automated and manual mapping of typhoon‐triggered landslides from SPOT‐5 imagery. International Journal of Remote Sensing.

[25] Yalcin A, Reis S, Aydinoglu AC, Yomralioglu T (2011). A GIS-based comparative study of frequency ratio, analytical hierarchy process, bivariate statistics and logistics regression methods for landslide susceptibility mapping in Trabzon, NE Turkey. Catena.

[26] NASA Landsat Program, 2014, Landsat 8 OLI_TIRS, LC81320412014358LGN00, GeoCover, USGS, China, 24/12/2014.

[27] Goudie A (2013). Characterising the distribution and morphology of creeks and pans on salt marshes in England and Wales using Google Earth. Estuarine, Coastal and Shelf Science.

[28] Banerjee P, Ghose MK, Pradhan R (2018). Analytic hierarchy process and information value method-based landslide susceptibility mapping and vehicle vulnerability assessment along a highway in Sikkim Himalaya. Arabian Journal of Geosciences.

[29] Gilbert JT, Macfarlane WW, Wheaton JM (2016). The Valley Bottom Extraction Tool (V-BET): A GIS tool for delineating valley bottoms across entire drainage networks. Computers & Geosciences.

[30] McFeeters SK (1996). The use of the Normalized Difference Water Index (NDWI) in the delineation of open water features. International journal of remote sensing.

[31] Snehmani, Singh MK, Gupta RD, Bhardwaj A, Joshi PK (2015). Remote sensing of mountain snow using active microwave sensors: a review. Geocarto International.

[32] Guo G, Chen J, Li MH, Dang J (2013). Statistic relationship between slope gradient and landslide probability in soil slopes around reservoir. Journal of Engineering Geology.

